# Highly Selective Hydrogen Peroxide Production Using an AgPd-Based Electrocatalyst with Ultralow Pd Loading

**DOI:** 10.1021/acsomega.5c04823

**Published:** 2025-09-04

**Authors:** Eleilde S. Oliveira, Fellipe S. Pereira, Jaynne S. Martins, Felipe A. e Silva, Ana Alcântara, Liying Liu, João M. A. R. de Almeida, Pedro N. Romano, Auro A. Tanaka, Thenner S. Rodrigues, Marco A. S. Garcia

**Affiliations:** † Department of Chemistry, Federal University of Maranhão (UFMA), São Luís 65080-805, MA, Brazil; ‡ Instituto de Química, Universidade Federal do Rio de Janeiro (UFRJ), Rio de Janeiro 21941-909, RJ, Brazil; § Nanotechnology Engineering Program, Alberto Luiz Coimbra Institute for Graduate Studies and Research in Engineering, COPPE, Universidade Federal do Rio de Janeiro (UFRJ), Rio de Janeiro 21941-972, RJ, Brazil; ∥ Centro Brasileiro de Pesquisas Físicas, Rio de Janeiro 22290-180, RJ, Brazil; ⊥ LIPCAT (Laboratório de Intensificação de Processos e Catálise), Universidade Federal do Rio de Janeiro (UFRJ), Rio de Janeiro 21941-594, RJ, Brazil; # Campus Duque de Caxias, Universidade Federal do Rio de Janeiro (UFRJ), Rio de Janeiro 25245-390, Brazil

## Abstract

Efficient electrocatalysts are key to advancing H_2_O_2_ production via the oxygen reduction reaction (ORR), but challenges like high material costs and low efficiency hinder progress. Based on this, this study focuses on the development and evaluation of a bimetallic AgPd/C electrocatalyst with ultralow Pd loading (0.2 wt %) designed for the selective ORR, specifically targeting the production of H_2_O_2_. The catalyst was synthesized using a galvanic replacement method, combining Ag and Pd in a random atomic arrangement on Vulcan XC-72 carbon support to optimize the dispersion and conductivity of active sites. Electrochemical analyses revealed that the AgPd/C electrocatalyst exhibited remarkable selectivity for H_2_O_2_, maintaining selectivity above 75% within a defined potential range. This selectivity significantly outperformed the Vulcan XC-72 catalyst, which showed a notable decline over the same range. Furthermore, the AgPd/C catalyst displayed superior resistance to methanol crossover compared to commercial Pt/C electrocatalysts, demonstrating its potential for stable and efficient operation in methanol-fueled applications. The combination of Ag and Pd enhanced the selectivity for the two-electron ORR mechanism and reduced energy requirements for initiating electroreduction, making it a promising candidate for applications requiring high H_2_O_2_ selectivity, such as green chemical processes. Our findings suggest that the selective 2e^–^ ORR behavior arises from the surficial structure achieved via the galvanic replacement synthesis with ultralow Pd content.

## Graphical abstract

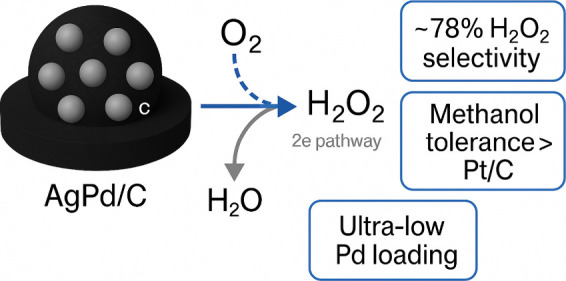

## 1 Introduction

The ORR, which can follow two- or four-electron transfer mechanisms, is crucial for energy conversion technologies such as fuel cells and metal-air batteries. However, it can be central to chemical processes such as producing hydrogen peroxide (H_2_O_2_), an environmentally friendly oxidizing agent.^1–4^ Among its important properties, H_2_O_2_ is used in chemical syntheses of organic and inorganic compounds, food processing, and wastewater treatments.^5–7^ In this sense, the two-electron ORR process is a safe alternative for H_2_O_2_ production, whose electrocatalytic process has been widely employed in research under alkaline conditions and with favorable performance using carbon-based catalysts.^8–10^ In this scenario, Platinum (Pt)-based electrocatalysts, while highly effective for catalyzing the ORR, are expensive and have limited availability, posing challenges to fuel cells’ widespread adoption and economic viability.^11–13^

Bimetallic NPs offer a promising alternative; incorporating two distinct metals into nanostructures makes associating their synergistic effects feasible, optimizing the electrocatalytic properties necessary for the ORR.^14^ This synergy often results in improved catalytic activity, stability, and tolerance to poisoning compared to monometallic catalysts. Thus, the design of these NPs involves selecting metals that can adjust the electronic structure and geometric configuration at the nanoscale, which can significantly enhance ORR kinetics.^15–18^ Recently, there has been a growing scientific interest in nanostructured bimetallic materials featuring partially oxidized configurations. These structures are advantageous due to their precise composition control, high metal utilization efficiency, and the tailored properties that result from their interactions.^19,20^

The literature explores using bimetallic electrocatalysts for the ORR through the galvanic replacement synthesis technique, which relies on the spontaneous redox reaction between a less noble metal core and a more noble metal precursor in solution, enabling the partial dissolution of the core metal and its simultaneous replacement by atoms of the other metal.^21–24^ The process induces atomic-scale randomness in the metals through combined kinetic and thermodynamic effects,^25,26^ producing surface compositions and electronic structures that modulate the d-band center of the surface atoms; these changes critically influence the adsorption energy of oxygen species, thereby enhancing catalytic interactions at the surface.^27^ In this sense, Ag has attracted attention as a sacrificial metal due to its abundance and cost-effectiveness. However, despite these advantages, its catalytic performance for ORR still falls short of Pt in alkaline conditions,^28^ bringing Pd into focus.

With physicochemical properties similar to Pt and greater electrochemical stability, reflected in its higher corrosion resistance compared to many other transition metals, Pd shows promise as a viable Pt substitute in electrocatalysis. Also, its low binding affinity with oxygen-containing species and reduced susceptibility to anion poisoning in alkaline solutions is highly interesting.^29,30^ Some of us have demonstrated that Ag and Pt systems, obtained via galvanic replacement reactions, are highly promising for the ORR due to the random surficial structure of the metals.^25,26^ We have also shown that, in Ag–Pd systems, varying the Pd content can lead to distinct ORR pathways.^31^ Furthermore, reports on nonelectrochemical H_2_O_2_ generation have highlighted the effective use of Ag–Pd nanostructures, suggesting that this bimetallic system holds unexploited potential for broader exploration in electrochemical processes.^32–34^

Based on this, our research explores the impacts of a bimetallic system comprising Ag and Pd, distinguished by an ultralow loading of the noble metal (0.2 wt % Pd), focusing on a 2-electron mechanism for ORR. Importantly, we employed the galvanic replacement process to achieve a random dispersion of both metals, without producing a hollow structure. This substantial reduction in Pd content not only preserves electrocatalytic performance and selectivity for H_2_O_2_ generation but also offers a clear economic advantage. Given the high cost and limited availability of Pd, this low-Pd catalyst represents a more sustainable and scalable solution for decentralized H_2_O_2_ production and electrochemical energy conversion applications. The bimetallic nanostructure was then immobilized on Vulcan XC-72 carbon, selected for its high surface area and excellent electrical conductivity. These characteristics facilitate the uniform dispersion of the NPs, enhancing their effectiveness. Our analysis demonstrated that this bimetallic structure provided selective active sites for ORR and displayed exceptional conductivity, offering promising insights for our research. Also, the prepared electrocatalyst proved to be more resistant to the methanol crossover effect than the Pt/C commercial electrocatalyst, which paves the way for possibilities. These findings show that we can provide a good performance of ORR in a Direct Methanol Fuel Cell (DMFC) configuration, with less methanol interference, still providing H_2_O_2_ that could be useful for other applications using ultralow Pd loadings.

## 2 Experimental Section

### 2.1 Materials and Instrumentation

All the reagents, which were of analytical grade, were purchased from Sigma-Aldrich and utilized as received. The chemicals used in our experiments included palladium­(II) chloride (PdCl_2_, ≥99.9%), silver nitrate (AgNO_3_, 99%), ethylene glycol (99.8%), hydroquinone (C_6_H_6_O_2_, 99%), polyvinylpyrrolidone (PVP, molecular weights of 10,000 and 55,000 g mol^–1^), and hydrochloric acid (HCl, 37%). The commercial Pt/C electrocatalyst and Vulcan XC-72 were procured from E-TEK. Pd/C electrocatalyst was purchased from Sigma-Aldrich. Imaging was performed by transmission electron microscopy (TEM) using a JEOL 2100F microscope (JEOL, Tokyo, Japan), operated at 200 kV and equipped with energy dispersive X-ray (EDX) spectroscopy. The sample for imaging was prepared by suspending the electrocatalyst in isopropanol, followed by drop-casting it onto a carbon-coated copper grid. Metal uptake onto the silica was quantified via atomic absorption spectrometry (AAS) using GFA-EX7i equipment (Shimadzu, Kyoto, Japan).

### 2.2 Synthesis of Ag Nanospheres

In a standard synthesis, 5.0 g of PVP (molecular weight 10,000 g mol^–1^) was dissolved in 37.5 mL of ethylene glycol. Subsequently, 200 mg of AgNO_3_ was introduced into the solution. Upon complete dissolution, the mixture was heated to 125 °C and maintained at this temperature for 2.5 h. The resultant solution, showing a greenish-yellow color, was then cooled to room temperature and diluted to a total volume of 125 mL with water.^35^

### 2.3 Synthesis of Bimetallic AgPd Nanostructures by a Galvanic Replacement Approach

To prepare AgPd NPs, 5 mL of a 0.1 wt % aqueous solution of PVP (molecular weight 55,000 g mol^–1^) was combined with 1 mL of the previously synthesized Ag nanospheres. This mixture was stirred at 100 °C for 10 min in a round-bottom flask. Then, dropwise, 2 mL of a 0.4 mM aqueous solution of the Pd precursor was added. The reaction was then allowed to proceed under stirring at 100 °C for 1 h.^36^

### 2.4 Immobilization of AgPd Nanostructures on Vulcan XC-72

The AgPd nanostructures were immobilized onto Vulcan XC-72 support using a wet impregnation method with modifications.^37,38^ For that, a suspension of AgPd NPs was poured into a beaker containing the support and stirred continuously for 24 h at room temperature. Subsequently, the resultant solid was sequentially washed twice with water and twice with ethanol. After washing, the catalyst was dried at 120 °C for 2 h under air to yield the AgPd/C electrocatalyst.

### 2.5 Pd/C Synthesis

The Pd/C electrocatalyst was prepared similarly to the previous AgPd/C counterpart; however, without the galvanic replacement step: 5.0 g of PVP (molecular weight 10,000 g mol^–1^) was dissolved in 37.5 mL of ethylene glycol. Subsequently, 209 mg of PdCl_2_ was added to the solution. After complete dissolution, the mixture was heated to 125 °C and maintained at this temperature for 2.5 h. The resulting solution was cooled to room temperature and diluted with water to a final volume of 125 mL. A portion of this solution, corresponding to a final Pd loading of 1.5 wt %, was combined with Vulcan XC-72 carbon and stirred for 24 h at room temperature. The solid was subsequently washed twice with water and twice with ethanol, then dried at 120 °C for 2 h in air to obtain the Pd/C electrocatalyst. AAS confirmed that the prepared catalyst achieved the target Pd loading (1.5 wt %).

### 2.6 Electrochemical Studies

For the electrochemical experiments, Autolab PGSTAT 302N and AUT302N.FRA32 M potentiostats (Metrohm, Herisau, Switzerland) were employed. The experimental arrangement included a three-electrode electrochemical cell consisting of a platinum foil auxiliary electrode, a reversible hydrogen electrode (RHE) as the reference, and a working electrode made of glassy carbon (GC), modified with the electrocatalyst. To prepare the catalyst suspensions, 1 mL of methanol with 0.1 mL of Nafion 5.0 wt %, 1.4 mL of deionized water, and 2.5 mg of the electrocatalyst were combined. The mixture was then ultrasonicated for 1 h. Before modification, the GC electrode was polished using alumina suspensions of varying granularity (1, 0.3, and 0.05 μm, supplied by Buehler Ltd., Illinois, USA). Afterward, 20 μL of each catalyst suspension was applied to the surface of the GC electrode and allowed to dry at room temperature.

The catalytic efficiency for the ORR in an alkaline solution (KOH 0.1 M) was evaluated using Cyclic Voltammetry, Linear Sweep Voltammetry, and rotating disk electrode methodologies. Additionally, the catalysts’ tolerance to methanol was assessed. The analysis also included generating Koutecky–Levich (K–L) plots utilizing the following equation:

$$\frac{1}{J}=\frac{1}{Jk}+\frac{1}{Jd}=\frac{1}{Jk}+\frac{1}{{Bw}^{1/2}}$$ 1

where ik is the kinetic current and id is the diffusion current, obtained through the Levich equation:

$$B=0,62nFADo^{2/3}\mathrm{Co}v^{-1/6}$$ 2

where *n* is the number of electrons transferred in the reaction, *F* is the Faraday constant equal to 96.485 C mol^–1^, *A* is the electrode area equal to 0.196 cm^2^, Co is the solubility of oxygen (1.103 × 10^–5^ mol cm^–3^), *D* is oxygen diffusion coefficient (1.76 × 10^–5^ cm^2^s^–1^), *v* is kinematic viscosity (1 × 10^–2^ cm^2^ s^–1^) and ω is the rotation speed at which the reaction occurs.

The selectivity of H_2_O_2_, usually represented by the mole fraction of H_2_O_2_ of the ORR products, the Faradaic Efficiency (EF), and the number of electrons transferred was calculated according to the measured disk current (ID) and the ring current (IR). *N* is the current collection efficiency of the Pt ring, which was determined to be 0.25.

H2O2(%)=200%×IrNID+IRN 3

$$\mathrm{EF}=\frac{i_{R}}{|i_{D}|N}\times 100$$ 4

n=4IDID+IRN 5

Tafel plots were obtained using the equation:

η=blog(j) 6

where η is the overpotential, *b* is the Tafel slope, and *j* is the current density.

In the Electrochemical Impedance Spectroscopy (EIS) studies, perturbation frequencies were set from 100 kHz down to the millihertz range, recording 10 points per decade with a signal amplitude of 10 mV_RMS_. The frequency perturbations were adjusted to range from 100 kHz to 0.01 kHz for assessments focused on the high-frequency domain. The chronoamperometry was carried out at a constant voltage of 0.77 V vs RHE in O_2_-saturated 0.1 M KOH solution at 43,200 s. This value is 0.1 V above the onset potential. Accelerated durability tests (ADT) were performed by first collecting initial activity. The electrode was then cycled from 1.06 to 0.566 V vs RHE at 100 mV/s in an oxygen-saturated electrolyte. Activity data was collected after 1000 cycles.1

## 3 Results and Discussion

Our research began with synthesizing an electrocatalyst containing Ag and Pd as the active components, using Vulcan XC-72 (carbon black) as the support. Our primary aim was to prepare a cheap material for ORR, aiming for H_2_O_2_ production. Based on our prior studies involving Ag and Pt or Ag and Pd, where a galvanic replacement method was utilized, we again opted to employ this technique.^26,31^ However, unlike our previous efforts, which targeted the formation of a hollow structure,^25^ our current objective was to achieve a random atomic arrangement with a solid structure. For this matter, we used spherical seeds of Ag followed by a low-concentration Pd precursor (0.4 mM) solution, which caused partial oxidation of the Ag species due to differences in their standard potentials. Afterward, the prepared AgPd NPs were immobilized by a wet-impregnation method on Vulcan XC-72. The final electrocatalyst was designed as AgPd/C.

Table 1 outlines the surface properties of the bare Vulcan XC-72 and after the immobilization of the AgPd NPs (AgPd/C electrocatalyst), focusing on surface area, pore volume, and pore diameter. Vulcan XC-72 exhibits a surface area of 263.32 m^2^ g^–1^; its pore volume is measured at 0.32 cm^3^ g^–1^, and the average pore diameter is 3.24 nm. When we look at the AgPd/C electrocatalyst, the surface area drops slightly to 232.80 m^2^ g^–1^, which is expected due to the immobilization process of the bimetallic NPs. The pore volume also decreases to 0.26 cm^3^ g^–1^, and the average pore diameter remains similar to Vulcan XC-72 at 3.28 nm. The small decrease in surface area and pore volume for the AgPd/C electrocatalyst will probably not affect the mass transport properties of the support after the immobilization. Despite this, the performance in catalytic applications will also be largely determined by the properties of the Ag and Pd NPs, such as their distribution, alloying, and interaction with the carbon support.

**Table 1:** Surficial Properties of the Support and the AgPd/C Electrocatalyst

material	surface area (m^2^ g^–1^)	pore volume (cm^3^ g^–1^)	pore diameter (nm)
Vulcan XC-72	263.32	0.32	3.24
AgPd/C	232.80	0.26	3.28

Thus, TEM and chemical mappings were performed to evaluate such properties. Figure 1A shows the TEM image of the prepared electrocatalyst. The darker spots in the image are the bimetallic NPs, while the lighter, more uniform background is the Vulcan XC-72 support. The expected quasi-spherical bimetallic nanostructures, with approximately 5 nm size (Figure S1), seem well-dispersed without significant agglomeration. Following, chemical mappings were performed to evaluate the metal distribution in the nanostructures. As expected, the carbon mapping is well distributed once it is the catalytic support (Figure 1B). Figure 1C,D show that both metals are located in the same sites, showing the successful galvanic process. There is some variation in the intensity of the spots, which indicates differences in the quantity of the material at those points. The more intense the spot, the higher the element concentration in that area. Therefore, AA analyses revealed the atomic composition of the electrocatalyst, showing that Ag and Pd were present in very different amounts, as anticipated, with atomic percentages of 86 at % for Ag and 14 at % for Pd, respectively.

**Figure 1:**
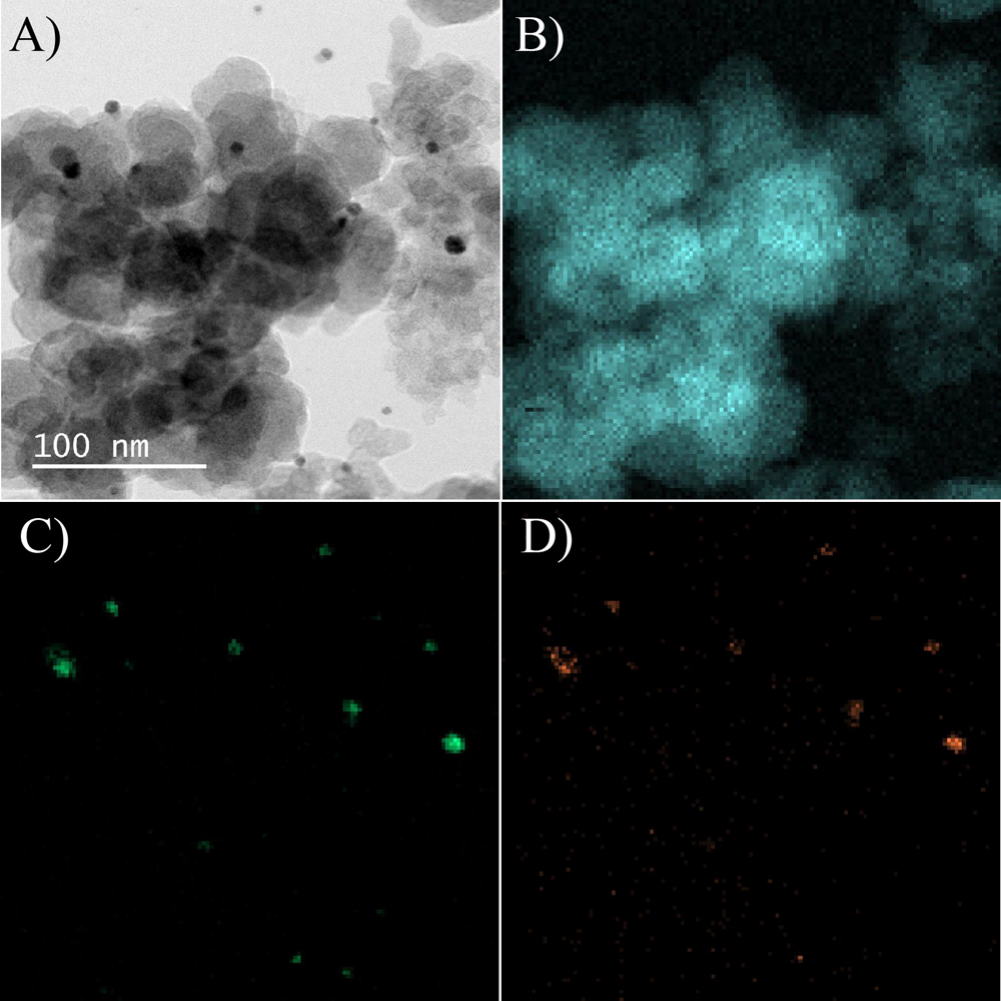
(A) TEM image of the AgPd/C electrocatalyst and chemical mappings of (B) carbon, (C) silver, and (D) palladium.

Furthermore, the loading of Pd on the support material was quantified by AAS at 0.2 and 1.25 wt % Ag. Aiming to understand the surficial metallic species of the electrocatalyst, high-resolution XPS analyses were performed. We examined the results based on Pd and Ag binding energies and identified 83.8% Ag^0^ and 16.2% Ag^+^ species (Figure 2A) and 54.0% Pd^0^ and 46.0% Pd^2+^ species (Figure 2B).^31,39^ Such results agree with the expectation that the galvanic replacement process was planned to occur in a controlled manner. Additionally, binding energy shifts could be explained by electronic interactions between Ag and Pd atoms at the surface, reflecting charge redistribution effects.

**Figure 2:**
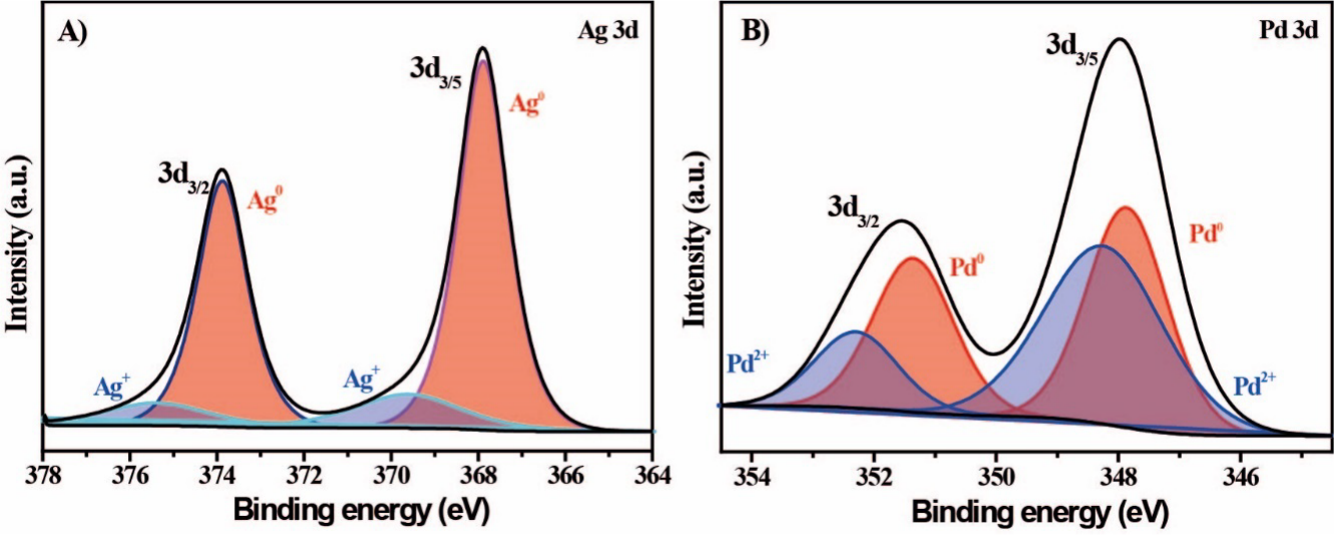
High-resolution XPS of (A) Ag 3d and (B) Pd 3d.

Cyclic voltammetry was used to characterize the AgPd/C electrocatalyst and obtain its electrochemical profiles. The nominal area of the working electrode was used to normalize these profiles. Figure 3A shows the electrochemical profiles in a solution of 0.1 M KOH saturated with N_2_ (black line) and O_2_ (red line). In both cases, electrochemical events in the range of 0.2–1.7 V vs RHE and a peak close to 1.5 V vs RHE are observed and associated with the formation of oxides on the surface of AgPd. A second peak, between 1.0 and 1.2 V vs RHE, indicates the reduction of these oxides formed due to the presence of Ag atoms in the electrocatalyst. In addition, a peak from approximately 0.9 V vs RHE can be attributed to the reduction of Pd­(II) oxides and the regeneration of metal Pd.^31^ When exposed to an O_2_-saturated electrolyte, the catalyst exhibits an excellent reducing response, later confirmed in ORR studies.

**Figure 3:**
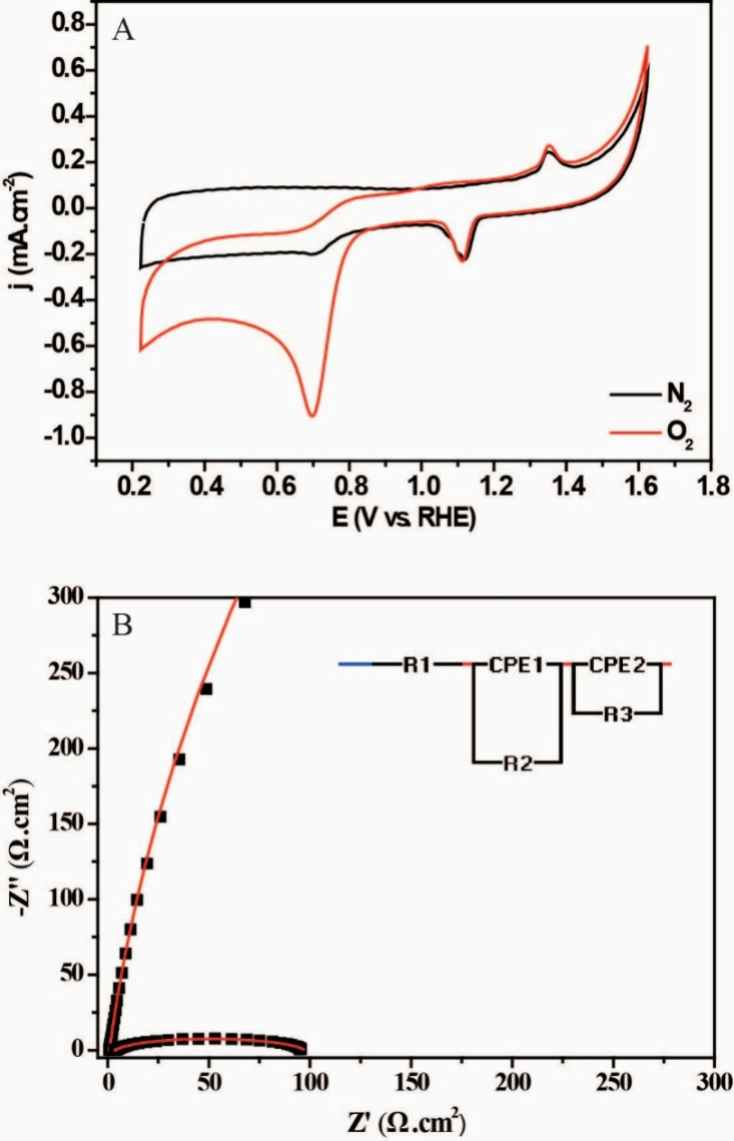
(A) CV curve of the AgPd/C electrocatalyst at a scan rate of 50 mV s^–1^ in N_2_ and O_2_-saturated 0.1 M KOH solution in the potential window of 0.23–1.62 V vs RHE. (B) EIS spectra for different regions of the ORR; the inset presents more details.

Following this, we chose to use EIS to evaluate the efficacy of the electrocatalyst in ORR. Figure 3B shows the EIS spectra for different regions of the oxygen reduction processes. In addition, Table 2 presents the parameters of the equivalent circuits for the AgPd/C electrocatalyst. Initially, the observed arc tends to converge to a semicircle for the nonpolarized electrode. When polarizing the electrode at cathodic potentials to induce the reaction of reduction of oxygen molecules, the formation of the semicircle and the decrease of its respective diameter are observed. Therefore, as the diameter of the semicircle indicates the *R*_ct_ of the electrochemical process,^40^ we observed a reduction in the load transfer resistance, as confirmed by the parameter values obtained when performing the impedance spectra adjustments. These results suggest that the material exhibits excellent catalytic activity toward ORR, indicating a low energy requirement to overcome the activation barrier and initiate electroreduction and high current density. These characteristics indicate an efficient electrochemical response of the material toward ORR.

**Table 2:** Equivalent Circuit Parameters for the AgPd/C Electrocatalyst at Different Regions of the Oxygen Reduction Process

potentials (V)	*R*_s_ (Ω)	*R*_ct_ (Ω)
0.2	35.0	1.46 × 10^5^
0.1	36.7	938

The rotating disc-ring electrode (RRDE) measurements for the AgPd/C, Pd/C, and Ag/C and Vulcan XC-72 electrocatalysts in a solution of KOH 0.1 M saturated-O_2_ at 1600 rpm, to evaluate their catalytic activity against ORR (Figure 4A) – data was normalized. Through the cathodic current density data, among the electrocatalysts, Pd/C showed the highest current density (3.65 mA cm^–2^), with an onset potential (*E*_o_) of 0.843 V vs RHE and a half-wave potential of (*E*_1/2_) 0.735 V vs RHE. The current density of the bimetallic electrocatalyst is 1.38 mA cm^–2^, whose *E*_o_ value (0.835 V vs RHE) is not significantly different. Still, it has more positive E1/2 (0.765 V vs RHE), indicating that the addition of Ag can confer a synergistic effect, potentially associated with the modification of the electron density and the creation of new active sites that favor O2 adsorption and electron transfer. However, it does not have the same levels of current density. On the other hand, Ag/C showed lower performance 0.43 mA cm^–2^), with *E*_o_ and *E*_1/2_ of 0.650 and 0.594 V vs RHE, showing less capacity to catalyze ORR efficiently. The Vulcan XC-72 presented expected values 1.59 mA cm^–2^), with *E*_o_ and *E*_1/2_ of 0.73 and 0.64 V vs RHE, confirming that the activity observed in the electrocatalysts containing Pd is associated with the supported metal. This unexpected trend likely arises from the combination of low silver loading (1.5 wt % Ag) and possible effects of the polyol synthesis route, which may influence dispersion on the support. Such factors can result in suboptimal active site exposure. Figure S2 shows the anodic currents (ring), for better observation.

**Figure 4:**
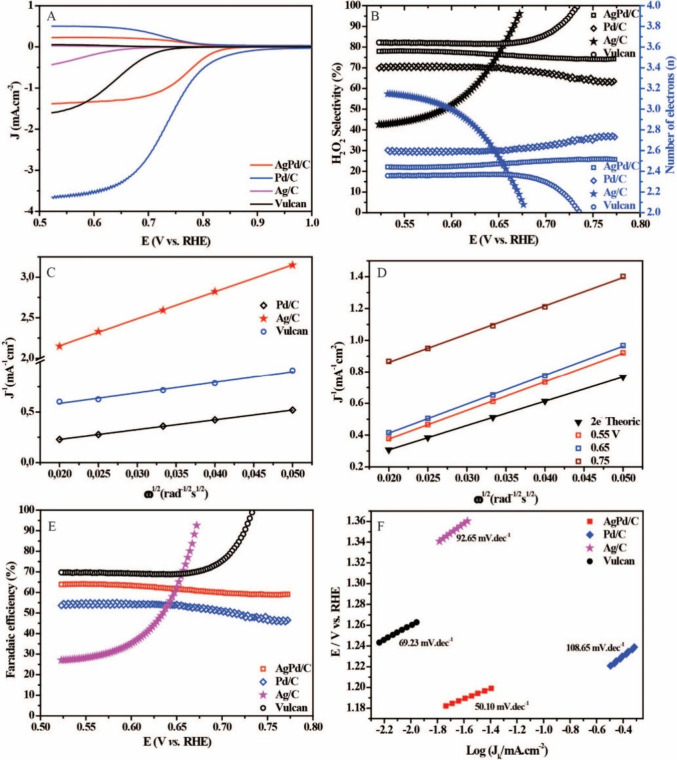
(A) LSV curves for the AgPd/C, Pd/C, Ag/C, and Vulcan electrocatalyst at a scan rate of 5 mV s^–1^ in O_2_-saturated 0.1 M KOH solution at 1600 rpm in the potential window of 1.0–0.52 V vs RHE (Ring potential 1.3 V vs RHE). (B) H_2_O_2_ selectivity and the number of electrons transferred for the electrocatalysts. (C) K–L plots for the AgPd/C in different potentials and (D) for the Pd/C, Ag/C, and Vulcan in 0.55 V vs RHE. (E) Faradaic efficiency of the electrochemical reduction of O_2_ to H_2_O_2_. (F) Tafel slopes of electrocatalysts.

Based on eqs 3 and 5, the data obtained by RRDE were used to calculate the number of electrons transferred (n) and their selectivity (H_2_O_2_%) during ORR to the different electrocatalysts (Figure 4B). In the case of AgPd/C, it is observed that the value of n varied slightly between 2.5 and 2.4 in the potential range between 0.77 and 0.52 V vs RHE, indicating that the process mostly follows a 2e^–^ mechanism. This behavior follows the high selectivity observed for the formation of H_2_O_2_, a characteristic product of the ORR two-electron pathway. Throughout the entire potential window studied, the selectivity for H_2_O_2_ remained practically constant, with values ranging between 74 and 78%, which evidence the stability of the catalytic activity of the material in promoting this reaction pathway. For the Pd/C, the n ranged from 2.7 to 2.58, and the selectivity was between 62 and 71%. These results indicate that, although the 2e_–_ pathway is still predominant, there is a more significant contribution of the 4e_–_ pathway compared to AgPd/C. Such behavior suggests that Pd/C has a greater capacity to promote complete oxygen reduction, although the partial formation of H_2_O_2_ remains relevant. For Ag/C, the variation of n was more significant, ranging from 2.0 to 3.1, whose selectivity followed this variation, ranging from 96 to 46%, a similar performance to pure Pd, but more abbreviated. Unlike AgPd/C, Vulcan obtained a more noticeable variation in both n and H_2_O_2_, from 2.0 to 24 and 99% to 81%, respectively, but still maintained its high ORR activity for the 2e^–^ mechanism.

Given these results, AgPd/C for its stable selectivity, combined with n values consistently close to 2e^–^. The presence of Pd, even in a smaller proportion, seems to give the system a catalytic synergy that maintains high selectivity without compromising stability, making AgPd/C a highly promising candidate for sustainable applications in H_2_O_2_ production. Evidence is also corroborated by the faradaic efficiency (eq 4) of the process (Figure 4C), whose variation did not exceed 5% for AgPd/C, while for Pd/C, Ag/C, and Vulcan, this variation occurred in 7, 65, and 29%, respectively.

As illustrated in Figure S3A–D, as observed for AgPd/C, Pd/C, Ag/C, and Vulcan electrocatalysts, a clear increase in ORR currents was observed as the rotational speeds of the disc electrode (RDE) were increased from 400 to 2500 rpm. This increase in current density with the increase in rotation speed is due to the shortening of the diffusion layer. Since the number of *n* reflects the catalyst’s efficiency in the process, it is crucial to determine it in the ORR. This parameter can also be obtained using the Koutecky–Levich equation (K–L), which analyzes the relationship between the bounded current (in mA) and the inverse square root of the rotational velocity (ω^–1/2^). In this study, *n*, as determined by linear regression of the K–L equation, was calculated from data on three selected potentials for the AgPd/C (Figure 4D). The resulting average value of 1.85 suggests that the electrocatalyst predominantly follows a two-electron pathway in reducing oxygen to hydrogen peroxide. Figure 4E shows the KL graphs for Pd/C, Ag/C, and Vulcan, whose *n* values were 3.17, 1.55, and 2.92, respectively. These results demonstrate that, while Pd/C does not have a specific pathway, Ag/C has an even lower *n* value, which indicates a limited catalytic activity and a less defined selectivity. Interestingly, the Vulcan stand itself displayed a value of *n* close to 3, likely associated with capacitive processes. Thus, among all the materials evaluated, AgPd/C stands out for presenting the most favorable combination between kinetic behavior and selectivity for the 2e^–^ mechanism. Its value of *n* close to 2, confirmed by both the RRDE analysis and the Koutecky–Levich equation, evidence its superior capacity to efficiently and stably catalyze the partial reduction of oxygen to H_2_O_2_.

Interestingly, this catalytic enhancement can be attributed to the polarization overpotential of Pd, which can reach up to 1.14 V, nearing the overpotential of Pt, which is around 1.38 V in alkaline solutions. Such proximity in polarization values enhances the adsorption of O_2_ molecules on the Ag surface, effectively improving the ORR kinetics. When combined with the inherent catalytic ability of Pd, these factors theoretically lead to superior reaction performance. Research by Qiu et al. has shown that with a relatively low Pd content in the alloy with Ag, the catalytic synergy primarily decreases from the predominant Pd–Ag bonding interactions. These interactions promote strong ligand and ensemble effects, significantly boosting the performance of ORR in alkaline media.^41^ The presence of Ag not only adjusts the electronic properties of the electrocatalyst but also alters the geometrical arrangement of atoms at the surface, further enhancing the electrochemical reduction process.^42^

The Tafel slopes for the ORR were calculated by plotting the current densities against the potential in the kinetically controlled transport region, as shown in Figure 4F, which confirmed that the AgPd NPs display rapid kinetics. Specifically, the Tafel slope values recorded were 50.10 mV dec^–1^ for AgPd/C, 108.65 mV dec^–1^, 92.65 mV dec^–1^, and 69.23 mV dec^–1^ for Vulcan XC-72. These values indicate the electrocatalytic efficiency, where lower Tafel slopes suggest faster ORR kinetics. Research has indicated that variations in Tafel slopes for Pd-based electrocatalysts typically result from differences in oxygen coverage on the catalyst surface, which do not negatively impact the ORR kinetics.^43^ In the case of the AgPd/C electrocatalyst, the presence of Ag enhances this dynamic. Silver’s strong adsorptive capacity likely prevents the excessive occupation of active sites on Pd, thus allowing more efficient catalysis of the oxygen reduction process. This effect maintains an optimal surface for the ORR and potentially enhances the overall activity by ensuring that palladium sites remain available for interaction with oxygen molecules. Furthermore, the integration of Ag in the bimetallic AgPd structure appears to modulate the electronic properties of Pd, optimizing the ORR process. This synergy between Ag and Pd in the AgPd/C electrocatalyst enhances the electrocatalytic reduction of oxygen.

Table 3 presents a comparative analysis of the AgPd/C electrocatalyst against several recently reported 2e^–^ ORR electrocatalysts for H_2_O_2_ generation in alkaline conditions. The AgPd/C developed in this work demonstrates a competitive onset potential (0.835 V vs RHE), which is close to the best-performing NiCo/CNT (0.85 V) and Ni-SAC (0.857 V), indicating favorable electrocatalytic activity for initiating the ORR. Despite its ultralow Pd loading (0.2 wt %), AgPd/C achieves 78% selectivity toward H_2_O_2_ with a near-ideal electron transfer number (*n* = 2.4), confirming that the 2e^–^ pathway is dominant. While its disk current density (1.38 mA cm^–2^) is lower than that of Ni-SAC (37.40 mA cm^–2^), it remains comparable to other materials with higher noble metal or dopant loadings, such as LP-C­(Fe)_2_(3:3) and VC/Nb/F composites.

**Table 3:** Comparison of AgPd/C with Reported Electrocatalysts for H_2_O_2_ Production via 2e^–^ ORR in Alkaline Medium^a^

electrocatalysts	*E*_onset_ (V vs RHE)	density current disk (mA cm^2^)	%H_2_O_2_	*n*	slope Tafel (mV dec^–1^)	reference
AgPd/C	0.835	1.38	78	2.4	50.10	this work
Ni-SAC	0.857	37.40	73	2.5	79.5	^45^
VC/Nb/F 1.0%		0.38 (mA)	80	2.4		^46^
LP-C(Fe)_2(3:3)	0.7	∼1.3	80	2.5		^47^
NiCo/CNT	0.85	2.49	∼90	∼2		^48^

a Parameters include *E*_onset_, disk current density, %H_2_O_2_ selectivity, electron transfer number (*n*), and Tafel slope.

Importantly, AgPd/C exhibits a notably low Tafel slope (50.10 mV dec^–1^), which indicates faster reaction kinetics compared to Ni-SAC (79.5 mV dec^–1^) and aligns well with the requirement for efficient and selective H_2_O_2_ generation. Although some PGM-free catalysts, such as NiCo/CNT, report even higher selectivity (∼90%), the AgPd/C system distinguishes itself by balancing selectivity, stability, and cost-effectiveness, particularly in the context of galvanic replacement synthesis, enabling ultralow noble metal content.

Long-term stability is a crucial factor in evaluating the electrocatalytic performance of catalysts. To assess the stability of AgPd/C, we conducted a chronoamperometry using the RRDE technique (0.77 V disc potential vs RHE and 1.4 V ring potential vs RHE), as shown in Figure 5A. After 43200 s of reaction, the ring current is approximately 72% of its initial value. However, the disk current decreased by about 60%, which can be attributed to the increase in the concentration of H_2_O_2_ in the electrolyte, negatively influencing the measurement of disk current. However, this significant decrease in current retention did not negatively interfere with H_2_O_2_ selectivity and reaction mechanism. Figure 5B shows that over the 43,200 s, % H_2_O_2_ increased from 58 to 86% and the mechanism of the reaction remained closer to that of 2e^–^, ranging from 2.82 to 2.27. These results show that throughout the evaluation period, there was a lot of H_2_O_2_ formation, which may have caused the loss of disc current. The faradaic efficiency in this period also corroborates with the selectivity data and *n*, showing that there was an increase of 41–76% in efficiency (Figure 5C). These results show that AgPd/C has good operational stability and maintains high selectivity for H_2_O_2_ production, even under prolonged operating conditions, being a promising candidate for sustainable electrochemical applications.

**Figure 5:**
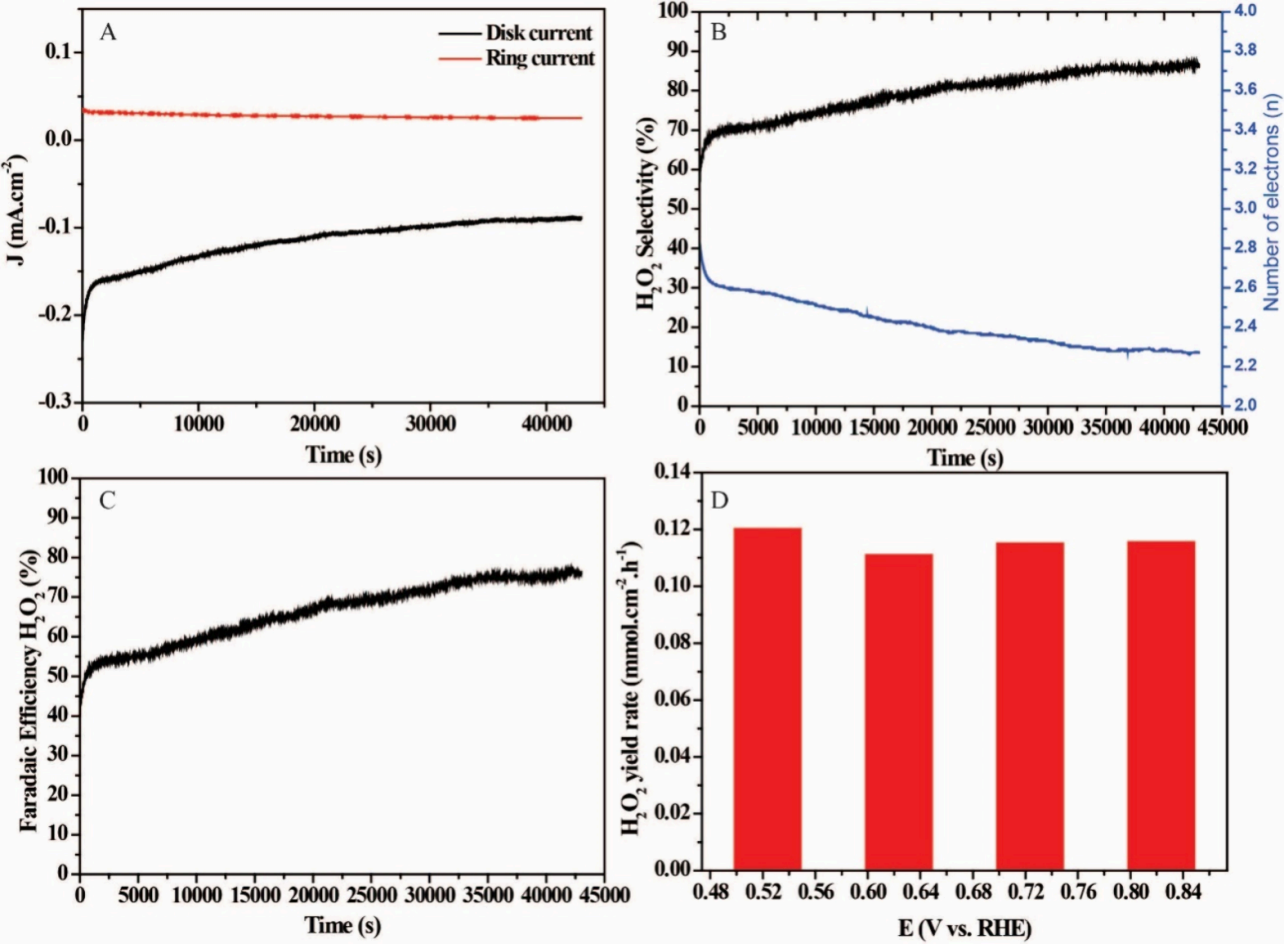
(A) RRDE chronoamperometry curve measured in O_2_-saturated 0.1 M KOH at 1600 rpm for 43,200 s of the AgPd/C. (B) Calculated H_2_O_2_ selectivity and (C) number of electrons (*n*) from RRDE chronoamperometry. (D) H_2_O_2_ yield rate (mmol cm^–2^ h^–1^) under alkaline conditions (0.1 M KOH).

To quantify H_2_O_2_, we used the cerium sulfate (Ce­(SO_4_)_2_) method to quantify the H_2_O_2_ production capacity. A solution of Ce­(SO_4_)_2_ was prepared at concentrations between 0.1 and 0.5 mM, using 0.5 M sulfuric acid as solvent. Then, a calibration curve relating the concentration of Ce^4+^ ions to the absorbance values obtained at 319 nm was performed using UV–vis spectrophotometry. After electrochemical experiments by the chronoamperometry technique at different potentials, a solution of Ce­(SO_4_)_2_ at 0.5 mM was added to the electrolyte. The variation in absorbance was monitored, considering that the yellowish Ce^4+^ ions react with hydrogen peroxide (H_2_O_2_), being reduced to Ce^3+^, a colorless species (eq 7). The difference in the concentration of Ce^4+^ before and after the addition of the electrolyte allowed us to estimate the amount of H_2_O_2_ formed, assuming the stoichiometry of the reaction, in which for each mole of H_2_O_2_ produced, two moles of Ce^4+^ are consumed, i.e., the concentration of H_2_O_2_ is equal to half the amount of reduced Ce^4+^.

$$2{\mathrm{Ce}}^{4+}H_{2}O_{2}\to 2{\mathrm{Ce}}^{3+}2H^{+}+O_{2}$$ 7

Based on these results for AgPd/C, the H_2_O_2_ yield rate remains without significant differences between the evaluated potentials, ranging from 0.12 to 0.11 mmol cm^–2^ h^–1^, as shown in Figure 5D. This stability indicates not only the efficiency of the material in the selective production of H_2_O_2_ but also its electrocatalytic robustness over the applied potential range. The constancy in the yield reinforces the good performance of AgPd/C in promoting the reaction via 2e^–^ efficiently, a desirable characteristic for applications in continuous processes of electrochemical generation of H_2_O_2_.

The ADT was performed to determine the durability of the AgPd/C electrocatalyst. This was by cycling the catalyst between 1.06 and 0.56 V vs RHE at a rapid rate at KOH 0.1 M saturated with oxygen. The results are presented in Figure S4A. The AgPd/C polarization curve before ADT exhibited lower activity when compared to the polarization curve after durability evaluation, as observed in disc and ring currents. An increase in selectivity was also observed regarding the formation of hydrogen peroxide, as shown in Figure S4B. In the window of potential work, from 1.06 to 0.56 V vs RHE, before ADT, the selectivity remained stable at 70% selectivity, while after the ADT, this selectivity had a significant increase, maintaining its selectivity at 80%. Such a phenomenon may occur due to the electrochemical activation process on the surface of the electrocatalyst and, thus, exposing more active sites, favoring the reaction via 2e^–^.

In the context of electrocatalysis for fuel cells, particularly in scenarios involving methanol as a fuel, the methanol crossover effect is a significant challenge that can drastically affect the efficiency and stability of the electrochemical reactions at the cathode. Figure S5 shows the CV profiles for two different electrocatalysts: the first is the AgPd/C electrocatalyst with a modest 0.2 wt % Pd loading (Figure S5A), and the second for a commercial Pt/C catalyst with a substantial 20 wt % Pt content (Figure S5B). Despite involving two metals, Pd and Pt, this comparison remains valuable as it illustrates our initial effort to contribute to the evolving field of substituting Pt with alternative metals requiring lower loadings. This attempt aligns with ongoing research to enhance the cost-effectiveness and sustainability of electrocatalysts by reducing reliance on Pt.

Figure S5A reveals how the AgPd/C electrocatalyst, despite being doped with a relatively low amount of Pd, demonstrates a certain resilience to the methanol crossover effect. Although methanol concentrations have a noticeable impact on the performance of the electrocatalyst, as evidenced by the shifts in current density with increasing methanol concentration, the effect is comparatively mild, which suggests that the AgPd/C electrocatalyst, while experiencing some degree of methanol crossover, does so at a significantly lower magnitude than might be expected given its metal content.

In contrast, Figure S5B, showing the commercial Pt/C electrocatalyst, exhibits a more pronounced influence of methanol on the electrocatalytic activity. Probably, the higher Pt loading in the Pt/C catalyst makes it more susceptible to methanol crossover effects. However, the incidence of 3 Pt sites near each other is also an issue in this case.^44^ Thus, the AgPd/C electrocatalyst, with its lower Pd content and effective alloying with Ag, shows a much lower sensitivity to methanol crossover than the commercial Pt/C material, suggesting a potential advantage in terms of operational stability and efficiency in methanol-fueled applications.

## 4 Conclusions

Our research successfully developed a bimetallic AgPd/C electrocatalyst with ultralow Pd loading (0.2 wt %) that demonstrated high selectivity for hydrogen peroxide production via the two-electron ORR pathway. The synergistic combination of Ag and Pd, facilitated by the galvanic replacement method, resulted in an electrocatalyst with well-dispersed active sites, excellent conductivity, and enhanced stability. The AgPd/C electrocatalyst outperformed the Vulcan XC-72 in terms of selectivity and durability, maintaining consistent H_2_O_2_ production across various potentials while also demonstrating superior resistance to methanol crossover compared to the commercial Pt/C electrocatalyst. Although the Pd loading is not yet aligned with the typical requirements for full-scale device integration, this study establishes an effective electrocatalytic performance using minimal noble metal content. Although the Pd content in the present electrocatalyst is ultralow, Pd remains a precious and scarce metal, which may still impose limitations on large-scale deployment; thus, our approach should be viewed as a step toward, but not a complete solution for, eliminating precious metal dependence in practical catalyst design. Also, we acknowledge that confirming the practical H_2_O_2_ production rate, concentration, and faradaic efficiency in bulk electrolysis setups such as H-type cells or flow reactors is essential for future applications. These experiments are beyond the scope of the present study but will be explored in our forthcoming work to validate the catalyst’s performance under practical operating conditions.

## Supplementary Material



## Data Availability

No data sets were generated or analyzed during the current study.

## Notes

Histogram of the NPs; LSV anodic currents (ring); LSV at different rotating speeds; polarization curves (disk and ring currents); and methanol crossover effect (PDF)
