# Supplementary material for: Highly Selective Hydrogen Peroxide Production Using an AgPd-Based Electrocatalyst with Ultralow Pd Loading
Source: ACS Omega. 2025 Sep 4;10(42):49710–20. doi: 10.1021/acsomega.5c04823 (PMC12572974; doi:10.1021/acsomega.5c04823)
Supplement: Supplementary file 1 [file ao5c04823_si_001.pdf]

Supporting Information

Highly Selective Hydrogen Peroxide Production Using An AgPd-based Electrocatalyst with Ultra-Low Pd Loading

Eleilde S. Oliveira,[a] Fellipe S. Pereira,[b] Jaynne S. Martins,[a] Felipe A. e Silva,[c] Ana Alcântara,[a] Liying Liu,[d] João M. A. R. de Almeida,[a,e] Pedro N. Romano,[e,f] Auro A. Tanaka,[a] Thenner S. Rodrigues,[c,*] Marco A. S. Garcia [a,c,e,*]

[a]Department of Chemistry, Federal University of Maranhão (UFMA), São Luís 65080-805, MA, Brazil.

[b]Instituto de Química, Universidade Federal do Rio de Janeiro (UFRJ), Rio de Janeiro 21941-909, RJ, Brazil.

[c]Nanotechnology Engineering Program, Alberto Luiz Coimbra Institute for Graduate Studies and Research in Engineering, COPPE, Universidade Federal do Rio de Janeiro (UFRJ), Rio de Janeiro 21941-972, RJ, Brazil.

[d]Centro Brasileiro de Pesquisas Físicas, Rio de Janeiro 22290-180, RJ, Brazil.

[e]LIPCAT (Laboratório de Intensificação de Processos e Catálise), Universidade Federal do Rio de Janeiro (UFRJ), Rio de Janeiro 21941-594, RJ, Brazil.

[f]Campus Duque de Caxias, Universidade Federal do Rio de Janeiro (UFRJ), Rio de Janeiro 25245-390, Brazil.

*thenner@pent.coppe.ufrj.br, marcosuller@pent.coppe.ufrj.br

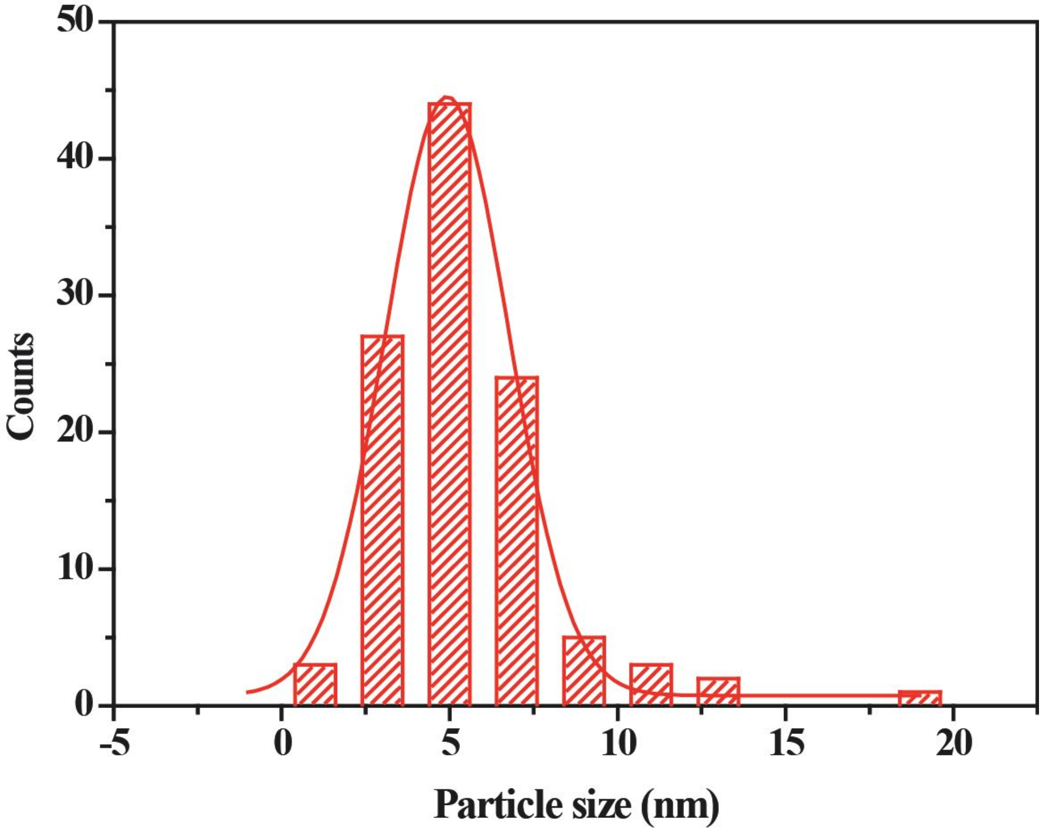

Figure S1. Histogram of the AgPd/C electrocatalyst.

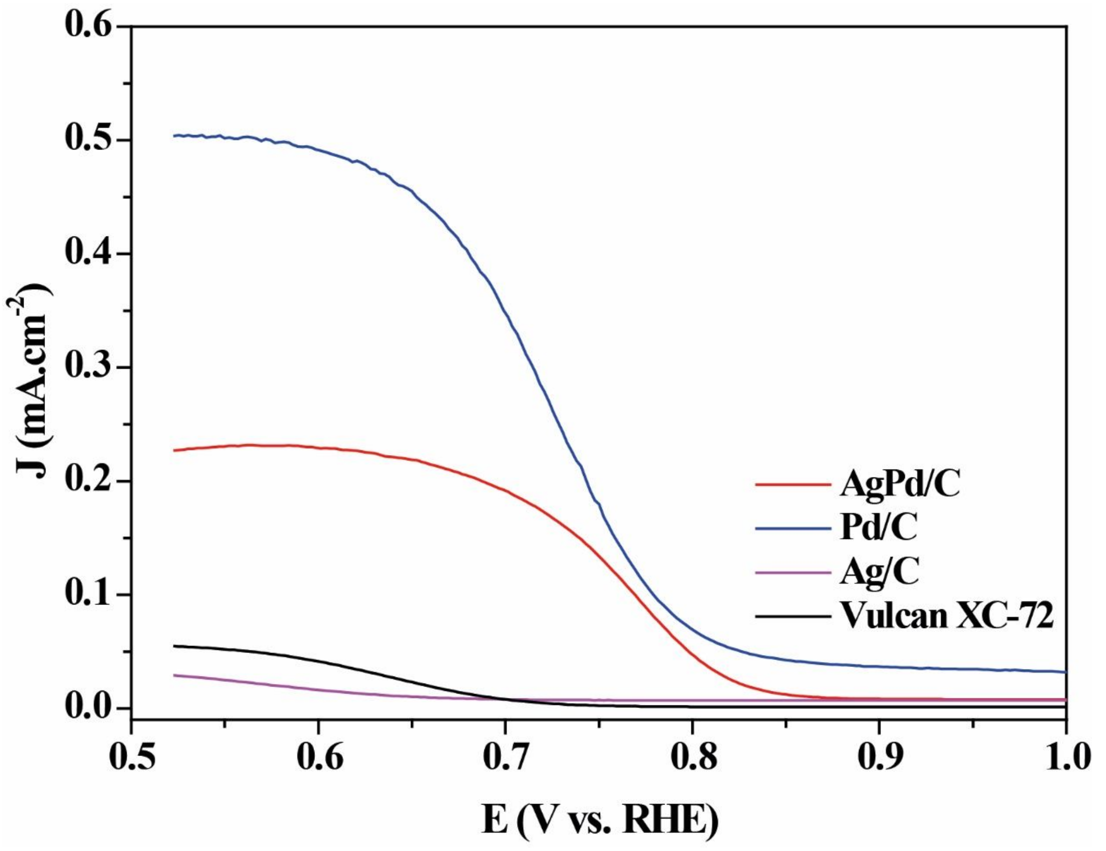

Figure S2. LSV anodic currents (ring).

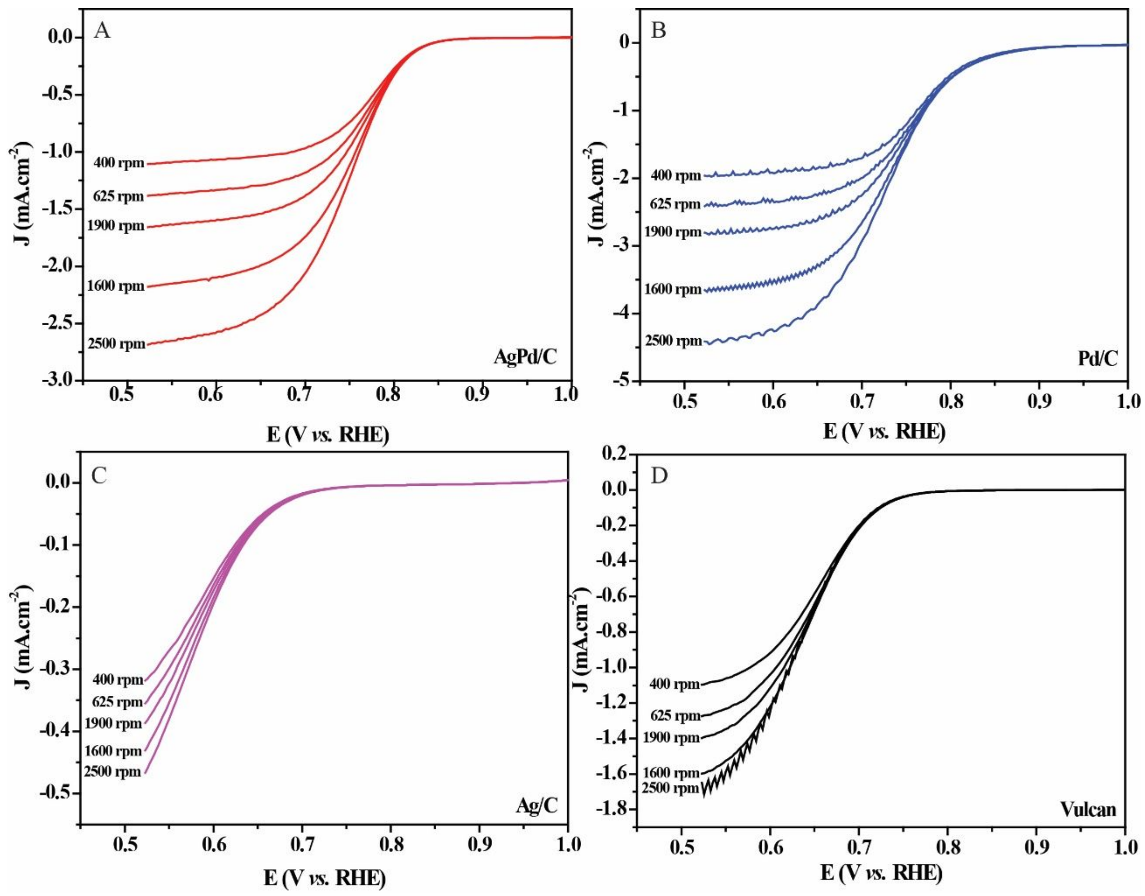

Figure S3. LSV at different rotating speeds recorded in O_2-saturated 0.1 M KOH electrolyte for A) AgPd/C, B) Pd/C, C) Ag/C, and D) Vulcan XC-72 electrocatalysts.

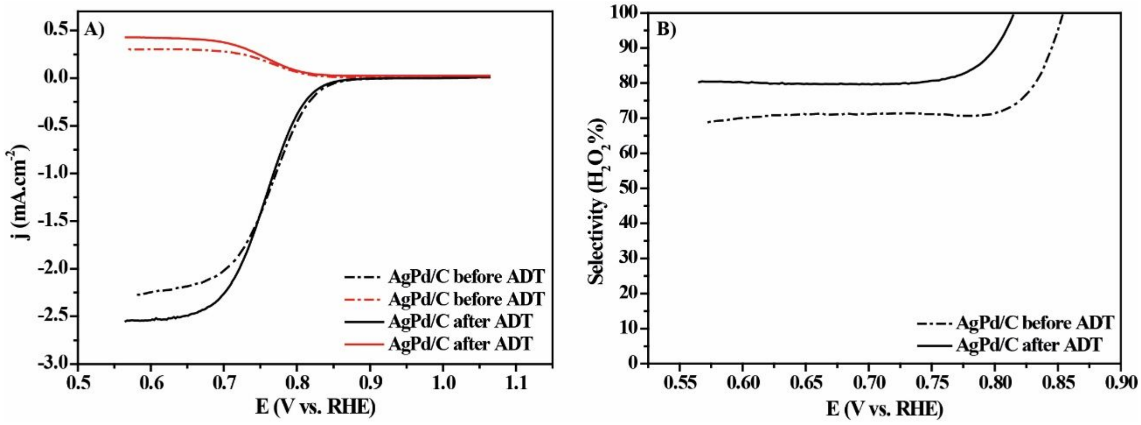

Figure S4. A) Polarization curves (disk and ring currents) for AgPd/C before and after the Accelerated Durability Test (ADT). B) Corresponding H_2O_2 selectivity profiles before and after ADT.

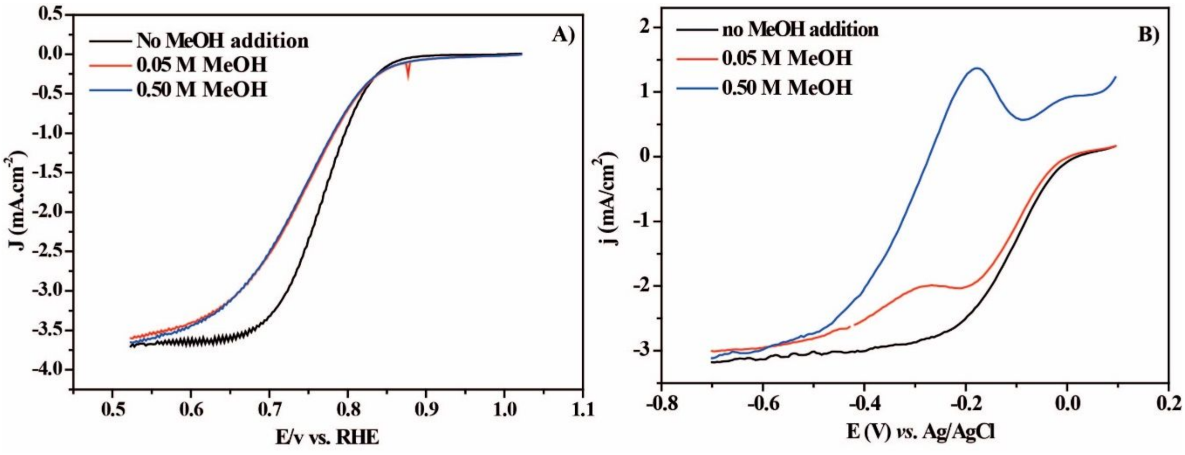

Figure S5. Methanol crossover effect comparison between A) AgPd/C (0.2 wt.% PD) and B) Pt/C (20 wt.% Pt) electrocatalysts.
